# Supplementary material for: Asymmetric bubble-mediated gas transfer enhances global ocean CO2 uptake
Source: Nat Commun. 2025 Nov 25;16:10595. doi: 10.1038/s41467-025-66652-5 (PMC12658222; doi:10.1038/s41467-025-66652-5)
Supplement: Supplementary file 1 — Supplementary Information [file 41467_2025_66652_MOESM1_ESM.pdf]

Supplementary Information for

Asymmetric bubble-mediated gas transfer enhances global ocean CO_2 uptake

Yuanxu Dong[1,2*], Mingxi Yang[3], Thomas G. Bell[3], Christa A. Marandino[1], David K. Woolf[4]

[1]Marine Biogeochemistry Research Division, GEOMAR Helmholtz Centre for Ocean Research Kiel, Kiel, Germany

[2]Institute of Environmental Physics, Heidelberg University, Heidelberg, Germany

[3]Plymouth Marine Laboratory, Plymouth, UK

[4]International Centre for Island Technology, Heriot-Watt University, Orkney, UK

[*]Corresponding to: ydong@geomar.de

1. K_{660_CO2} derived from the symmetric bulk equation

Rewriting of Equations 1 and 2 in the main text, making *K* the subject:

$$K = Flux/(C_w - C_a) \quad \text{(S1)}$$

$$K = Flux/[C_w - C_a(1 + \Delta_s)] \quad \text{(S2)}$$

As illustrated in the main text, the *K* derived from Equation S2 is the 'asymmetric' equation of transfer velocity ($K_{\_CO2\_asy}$), and derived from Equation S1 is the 'symmetric' equation of transfer velocity ($K_{\_CO2\_sy}$). Comparing Equations S1 and S2:

$$K_{\_CO2\_sy}/K_{\_CO2\_asy} = [C_w - C_a(1 + \Delta_s)]/(C_w - C_a) \quad \text{(S3)}$$

Normalizing *K* to K_{660}, replacing C_w and C_a by αfCO_{2w} and αfCO_{2a} respectively, and re-writing Equation S3:

$$K_{660\_CO2\_sy} = K_{660\_CO2\_asy}\left(1 - \frac{\Delta_s fCO_{2a}}{\Delta fCO_2}\right) \quad \text{(S4)}$$

Here, the 'asymmetric' equation of transfer velocity ($K_{660\_CO2\_asy}$) does not depend on ΔfCO_2, representing the unbiased K, while the 'symmetric' equation of transfer velocity ($K_{660\_CO2\_sy}$) is biased and depends on ΔfCO_2. Based on Equation S4, the $K_{660\_CO2\_sy}$ will be biased high for invasion cases (i.e., $\Delta fCO_2 < 0$) and biased low for evasion cases (i.e., $\Delta fCO_2 > 0$). Moreover, the bias will be stronger when $|\Delta fCO_2|$ is closer to zero (i.e., weak invasion and evasion) and wind speed is higher (Δ_s will be higher at high wind speeds). Therefore, we expect K_{660_CO2} derived from the symmetric bulk equation to have the order: weak invasion > strong invasion > strong evasion > weak evasion, especially at high wind speeds.

2. The theory of 2D fit

A two-dimensional (2D) fit method was used to make low $|\Delta fCO_2|$ observations available for analysis.

$$F = \Delta C_{660}\left(aU_{10N}^{b} + c\right) \quad \text{(S5)}$$

where $\Delta C_{660} = (C_w - C_i)\,(Sc/660)^{-0.5}$.

There are also differences between 1D and 2D fits in the weighting of data. The least-square fit works by finding the best coefficient with the lowest sum of the squares of the offsets (*SS*). For the 1D fit:

$$SS = \sum_{i=0}^{n}(K_{obs} - K_{1D\,fit})^2 \quad \text{(S6)}$$

Since K (and absolute errors in K) are generally high at high wind speeds, the 1D fit tends to be weighted towards those high wind conditions.

For the 2D fit:

$$SS = \sum_{i=0}^{n}[(K_{obs} - K_{2D\,fit})^2(\Delta C_{660})^2] \quad \text{(S7)}$$

The observations with low $|\Delta C_{660}|$ have weaker weight on the fit process, and the 2D fit tends

to be weighted towards high flux conditions. The calculated K values when the sea-air concentration difference is near zero (i.e., equilibrium) are weighted less, which is reasonable as their uncertainty is greater. Additionally, Fig. S2A indicates that the mean ΔfCO_2 (and consequently ΔC_{660}) remains relatively consistent across different wind speeds for each group, except in the case of strong invasion scenarios. This suggests that the 2D fit applies equal weighting under varying wind conditions, similar to the 1D fit. For the strong invasion scenario, the results of the 2D fit closely align with those of the 1D fit, with both approaches indicating lower K values compared to weak invasion scenarios and higher values compared to the strong evasion cases (Table S1).

To simplify things, all the fits shown in the main text are set with a zero constant (i.e., $K_{660} = a\, U_{10N}^{b} + 0$). We tried to fit with a non-zero constant, and the result does not change too much (See Fig. S13).

3. Re-analysis of the EC flux data

A method to estimate Δ_s and the asymmetric equation of transfer velocity (K_{CO2_asy}) is summarized here. The necessary inputs are:

- Processed EC fluxes (F), the derived gas transfer velocity based on the symmetric bulk equation (K), and CO_2 concentration at the subskin (C_w) and skin (C_a) depths.
- Overpressure factor value of δ, assumed to have a value of 0.0132 based on Leighton et al. (2018)[4].
- K_{int}, estimated using Blomquist et al. (2017)[1].
- f_{CE}, estimated chemical enhancement factor[1,2].

Following these input values, the steps are:

1) "guess" Δ_s from δ, K, and K_{int}. (Note that this takes K as a "zeroth" guess of K_{CO2_asy} to allow a first guess of Δ_s).
2) "guess" $K_{\_true}$ from F, C_w, C_a, and Δ_{s1} (where Δ_{s1} is the first guess of Δ_s).
3) Apply an iterative loop, re-guessing Δ_s and $K_{\_asy}$ sequentially until $K_{\_asy}$ approaches an asymptotic value.

Mathematically, the process is represented as follows:

- Set of values [F, K, C_w, C_a].
- $\delta = 0.0132$.
- $K_{int} = (Sc/\ 660)^{-1/2}\ (0.74U_{10}^{1.33})$ (see reference 1).
- $f_{CE} = K_{CE}/K - 1 = 18.12U_{10}^{-2.37}$ (see references 2, 3)

First guesses of Δ_s and $K_{\_asy}$:

$$\Delta_{s,1} = \delta(K - K_{int})/K \quad \text{(S8)}$$

$$K_{\_true,1} = F/\left[C_w - C_a\left(1 + \Delta_{s,1}\right)\right]/(1 + f_{CE}) \quad \text{(S9)}$$

Iterative loops of the form, $X_{i+1} = f(X_i)$, starting with $i = 1$.

$$\Delta_{s,i+1} = \delta\left(K_{\_asy,i} - K_{int}\right)/K_{\_asy,i} \quad \text{(S10)}$$

$$K_{\_asy,i+1} = F/\left[C_w - C_a\left(1 + \Delta_{s,i+1}\right)\right]/(1 + f_{CE}) \quad \text{(S11)}$$

Values of $K_{\_asy}$ and Δ_s should rapidly stabilize. The iterative loop stops when $K_{\_asy}$ changes by less than 0.001% per step. The dominant source of uncertainty is the value of δ, but the value from a recent study[4] is taken as a realistic value.

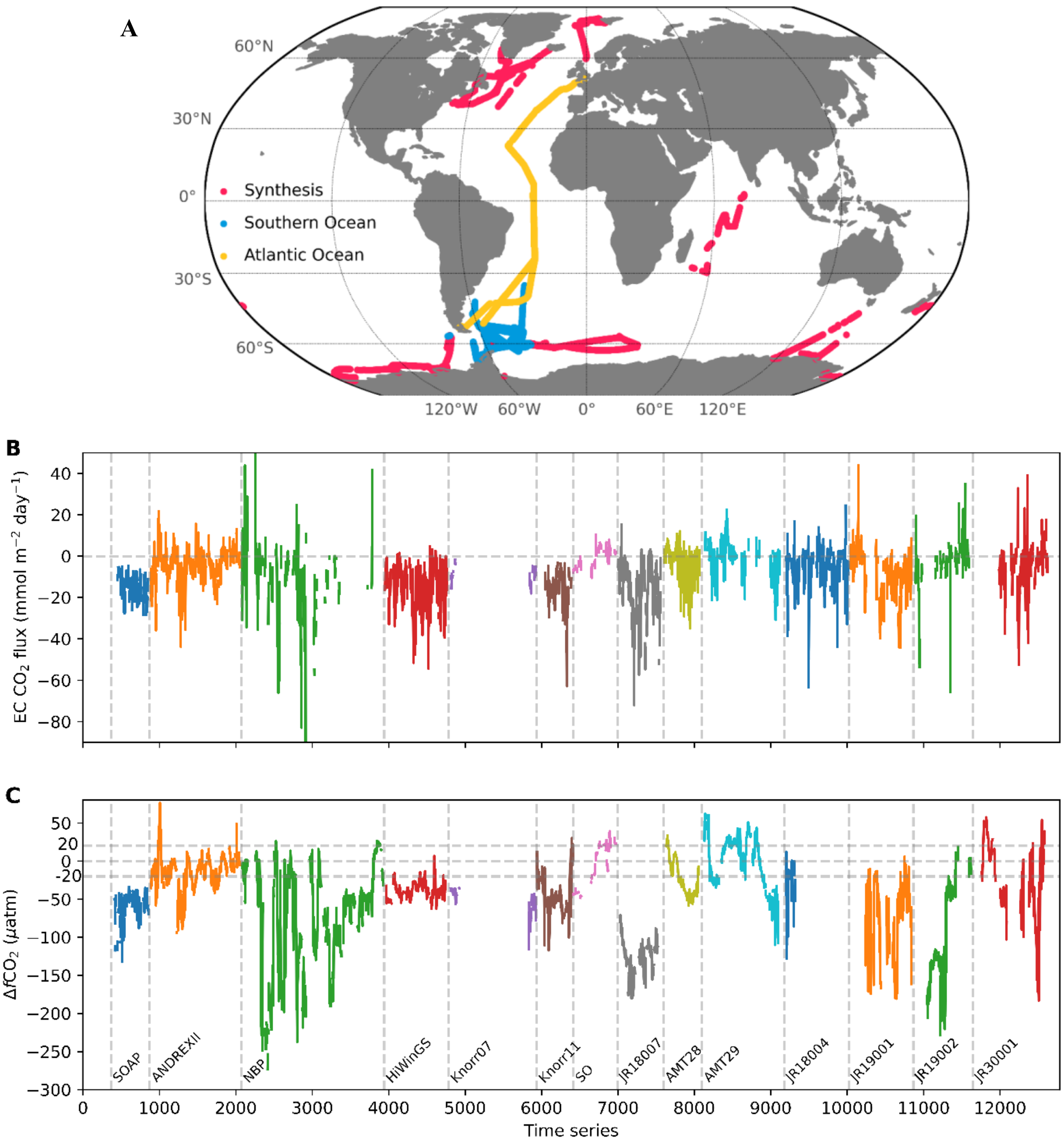

Fig. S1. Eddy covariance (EC) data collected from 17 cruises. A: Red lines represent 11 cruises from an EC CO_2 synthesis study[5], and see references therein for individual cruises, while yellow and blue lines denote additional datasets collected in the Atlantic Ocean (two cruises[6]) and Southern Ocean (four cruises[7]). **B** and **C**: Time series of EC flux and sea-air CO_2 fugacity difference (ΔfCO_2), respectively, from multiple cruises, with each cruise separated by vertical dashed lines. Cruise names are labeled above the bottom axis of panel C. The SOAP, ANDREXII, NBP (NBP-1210 and NBP-1402), HiWinGS, Knorr07 (Knorr07a and Knorr07b), Knorr11, and SO (SO-234 and SO-235) correspond to the 11 synthesized cruises (i.e., red lines in panel A). AMT28 and AMT29 represent the two cruises in the Atlantic Ocean (i.e., yellow lines in panel A), while the remaining four cruises are additional collections in the Southern Ocean (i.e., blue lines in panel A).

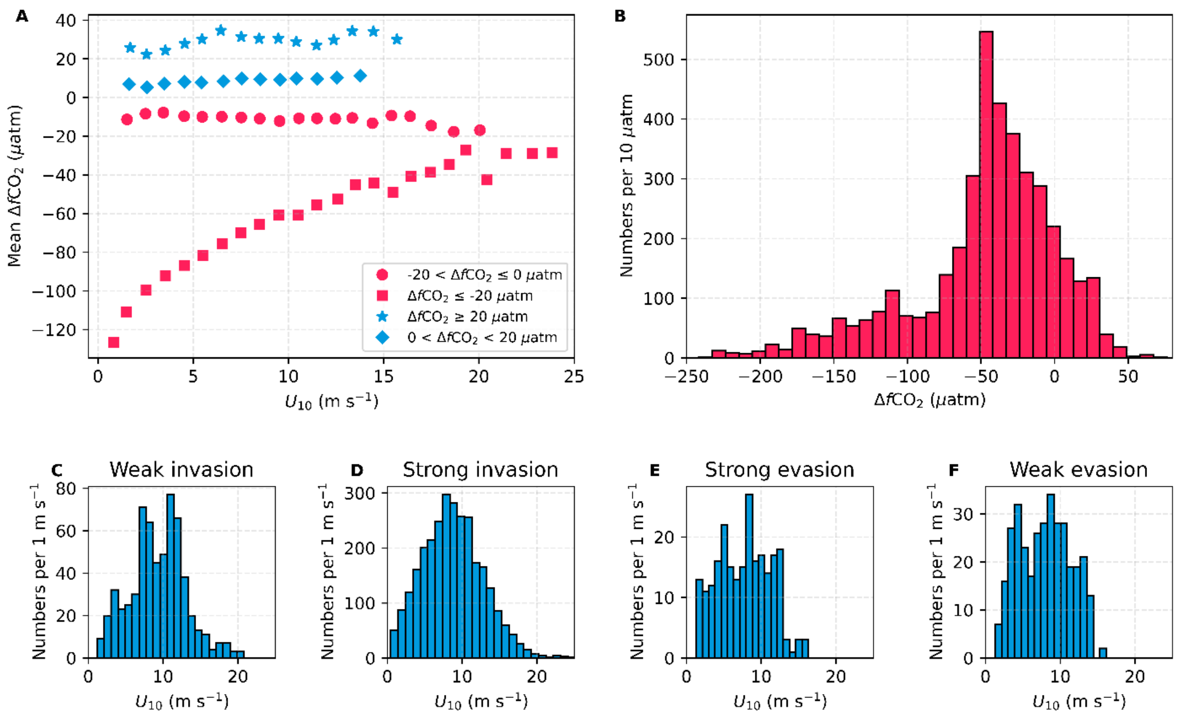

Fig. S2. Distributions of the data used in this study. A: Mean ΔfCO_2 versus wind speed (U_{10}) for four groups of data, with an averaging bin size of 2 m s^{-1} wind speed**.** Four data categories are shown: weak invasion (-20 < ΔfCO_2 ≤ 0 µatm, red dots), strong invasion (ΔfCO_2 ≤ -20 µatm, red squares), strong evasion (ΔfCO_2 ≥ 20 µatm, blue stars), and weak evasion (0< ΔfCO_2 < 20 µatm, blue diamonds). **B**: Histograms of the ΔfCO_2 for the data used in this study. **C-F**: Histograms of the U_{10} for the four data categories (defined the same as in panel A).

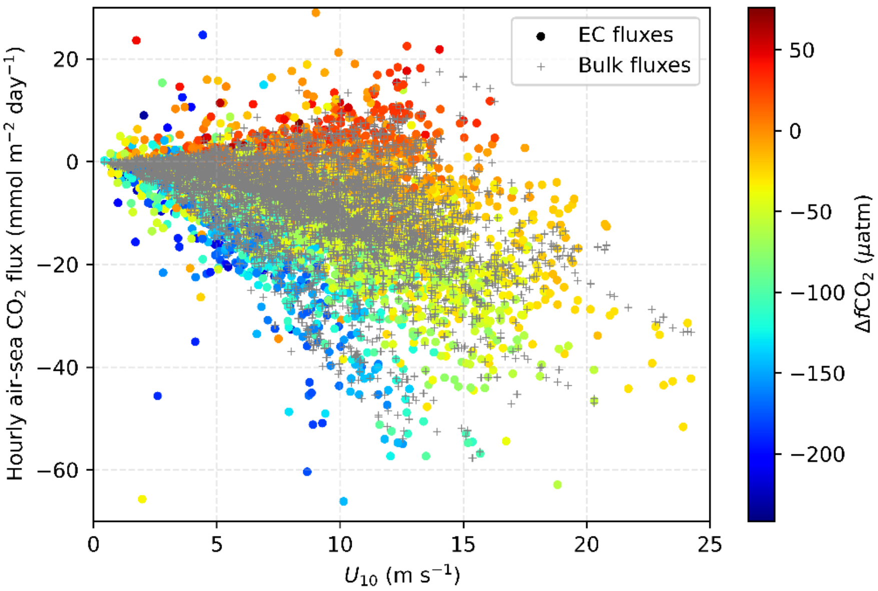

Fig. S3. Illustration of the 2D fit: hourly sea-air CO_2 flux observations and estimates versus U_{10}. The dots represent the EC sea-air CO_2 flux observations, color-coded by the ΔfCO_2. The crosses denote the bulk flux estimates using the asymmetric bulk equation (i.e., Equation 2 in the main text) and the 2D fit method.

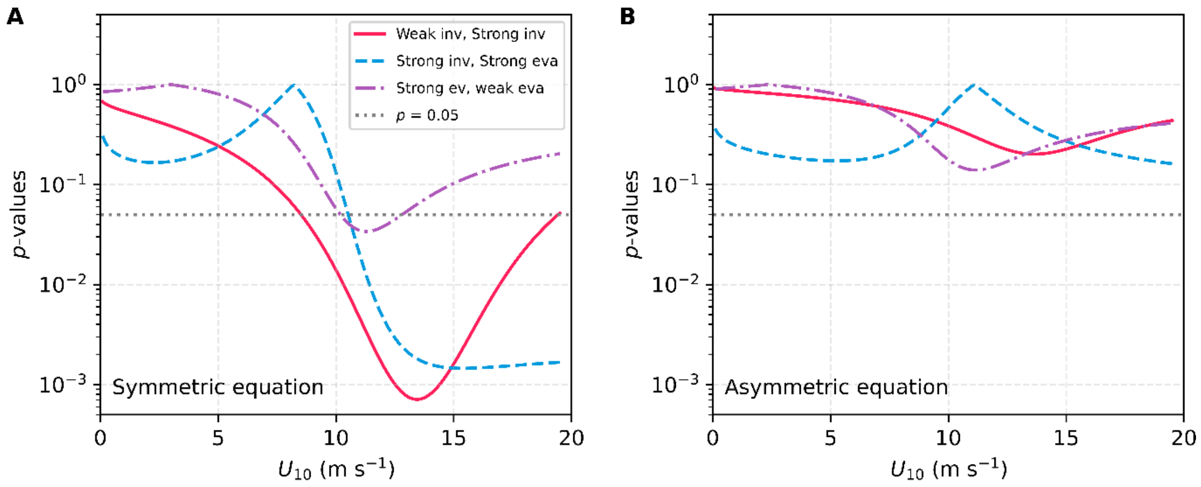

Fig. S4. *T*-test results (*p*-values) comparing different flux regime fits under the symmetric (A) and asymmetric (B) equations across a range of wind speeds. Red line: difference between weak invasion and strong invasion fits; Blue line: difference between strong invasion and strong evasion fits; Purple line: difference between strong evasion and weak evasion fits. Below the grey dashed line is significant ($p = 0.05$ threshold).

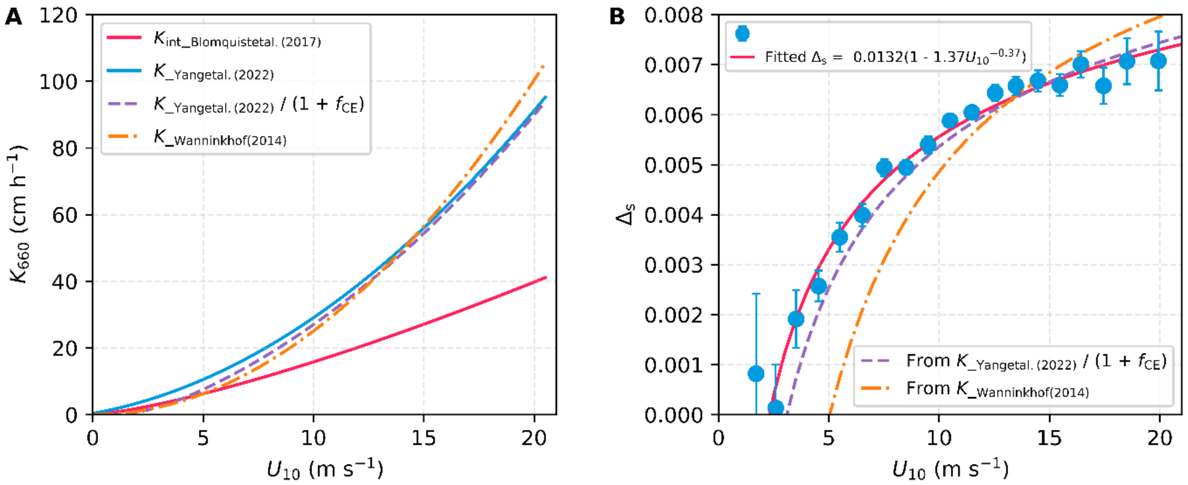

Fig. S5. Gas transfer velocity and constrained asymmetric factor (Δ_s) versus U_{10}. A: Interfacial transfer velocity (K_{int}) based on EC dimethylsulfide (DMS) observations[1] (red-solid line) and total transfer velocity (K) from the EC air-sea CO_2 flux observations[5] (blue-solid line) and from the ^{14}C inventory[8] (orange-dashed line). The EC-based parameterisation[5] with a chemical enhancement correction[2,3] (i.e., $K/(1 + f_{CE})$; here f_{CE} is parameterised with the U_{10} as $18.12U_{10}^{-2.37}$; see the text in section 3) is shown as the purple-dashed line. **B**: Δ_s constrained using different approaches. Blue dots represent 1 m s^{-1} binned averages of Δ_s derived from EC data reanalysis, with error bars indicating ± 1 standard error. The red line (Equation 5 in the main text, $R^2 = 0.11$) is a least-squares fit to the blue dots for wind speeds of 5-20 m s^{-1}. Data at $U_{10} < 5$ m s^{-1} are excluded from the fit because bubble-mediated and thus asymmetric transfer is expected to be negligible. The orange and purple dashed curves show the parameterised Δ_s based on the K formulation from ^{14}C inventory[8] and from EC[5] with a chemical enhancement correction, respectively.

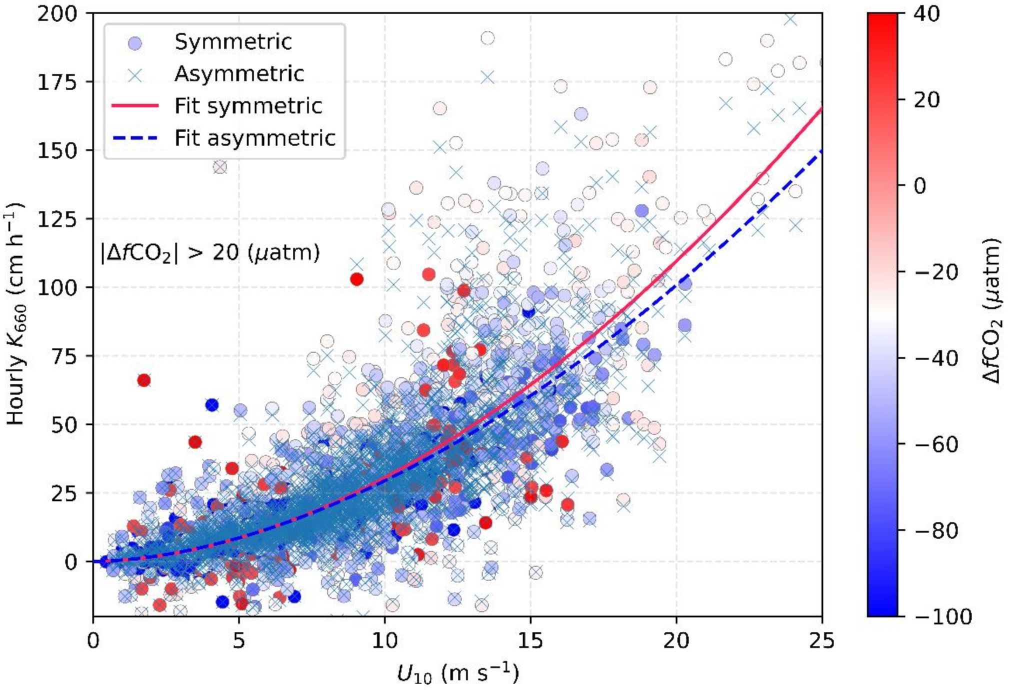

Fig. S6. Hourly CO_2 transfer velocities derived from EC sea-air CO_2 flux observations with the 1D method. Dots are calculated by the standard symmetric bulk equation (Equation 1, $K_{\_Sy}$), while crosses are calculated using the asymmetric bulk equation (Equation 2, $K_{\_Asy}$). The $K_{\_Sy}$ is colour-coded by ΔfCO_2. The gas transfer velocity has been normalized to the Schmidt number of 660, and only the data with $|\Delta fCO_2| > 20$ µatm are analyzed here. Red-solid line is the symmetric fit with the traditional 1D (one-dimension) method ($K^{1D}{}_{\_Sy} = 0.44U_{10}^{1.84}$, $R^2 = 0.59$), while the blue-dashed line is the asymmetric fit with the 1D method ($K^{1D}{}_{\_Asy} = 0.49U_{10}^{1.78}$, $R^2 = 0.58$).

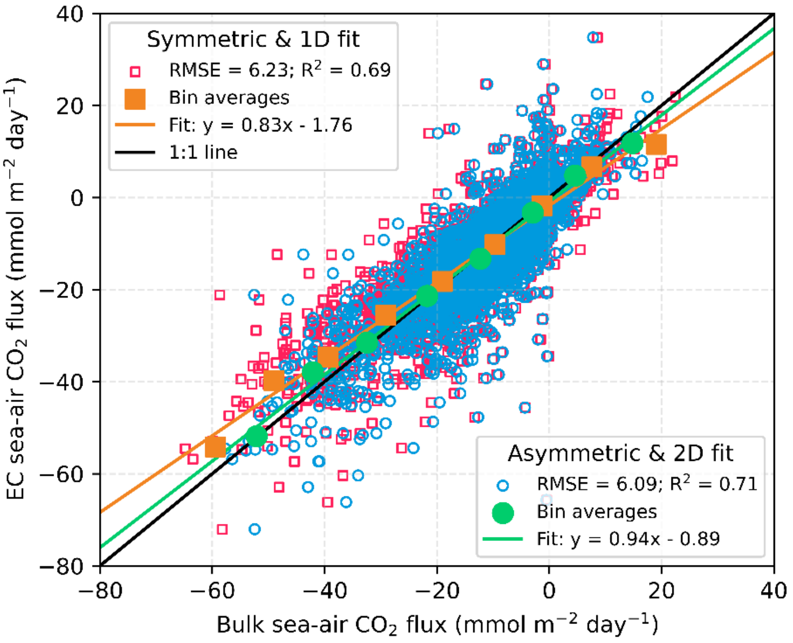

Fig. S7. Comparison of EC flux observations with bulk flux estimates. Red squares show fluxes estimated using the symmetric equation and *K* parameterisation from the 1D fit (red line in Fig. S6); orange squares and line indicate bin averages and the corresponding linear fit. The root mean square error (RMSE) and R^2 for the symmetric equation are 6.23 and 0.69, respectively. Blue dots show fluxes estimated using the asymmetric equation and *K* parameterisation from the 2D (two-dimension) fit (lines in Fig. 1B); green squares and line indicate bin averages and the corresponding linear fit. The RMSE and R^2 for the asymmetric equation are 6.09 and 0.71, respectively. It shows that the asymmetric formulation aided by the 2D fit yields a higher R^2, lower RMSE, and is closer to the 1:1 line.

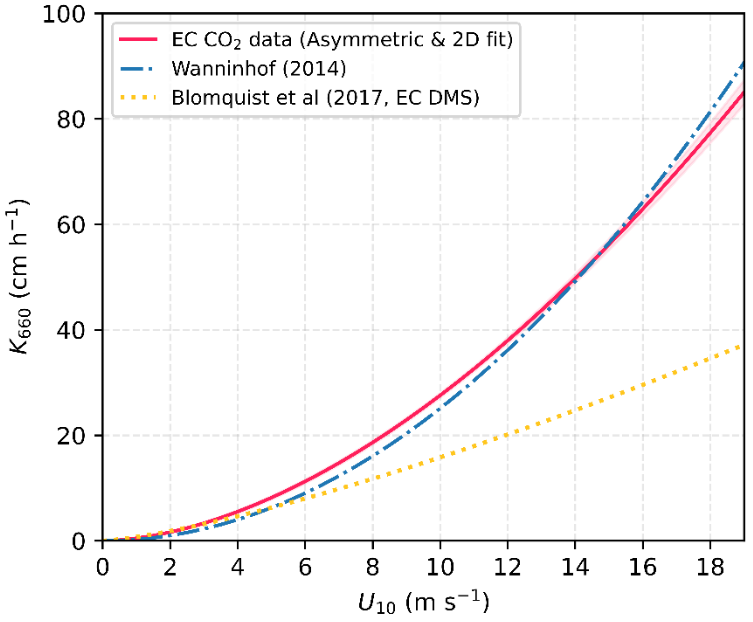

Fig. S8. Parameterisations of K_{660}-U_{10} based on different gas observations. Red line: the 2D fit to all the EC sea-air CO_2 flux data used in this study, with the red shadow representing the 95% confidence interval ($K^{2D}{}_{\_Asy} = 0.49U_{10}^{1.75}$, $R^2 = 0.71$). Blue-dot-dashed line: K_{660} based on global ^{14}C inventory[8]. Yellow-dashed line: K_{660_DMS} based on EC sea-air DMS flux observations[1], a proxy for interfacial gas transfer that assumes minimal bubble-mediated transfer of DMS.

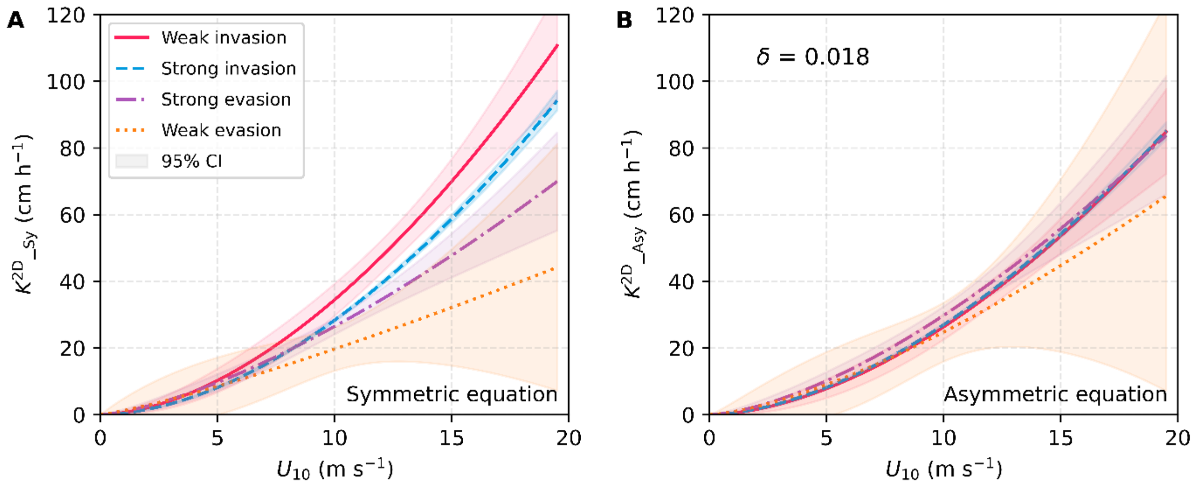

Fig. S9. Parameterisation of gas transfer velocity with U_{10}. The parameterisation of K^{2D} derived from eddy covariance (EC) observations with **(A)** the symmetric bulk equation (Equation 1), and **(B)** the asymmetric bulk equation (Equation 2) with the bubble over-pressure factor (δ) set as 0.018.

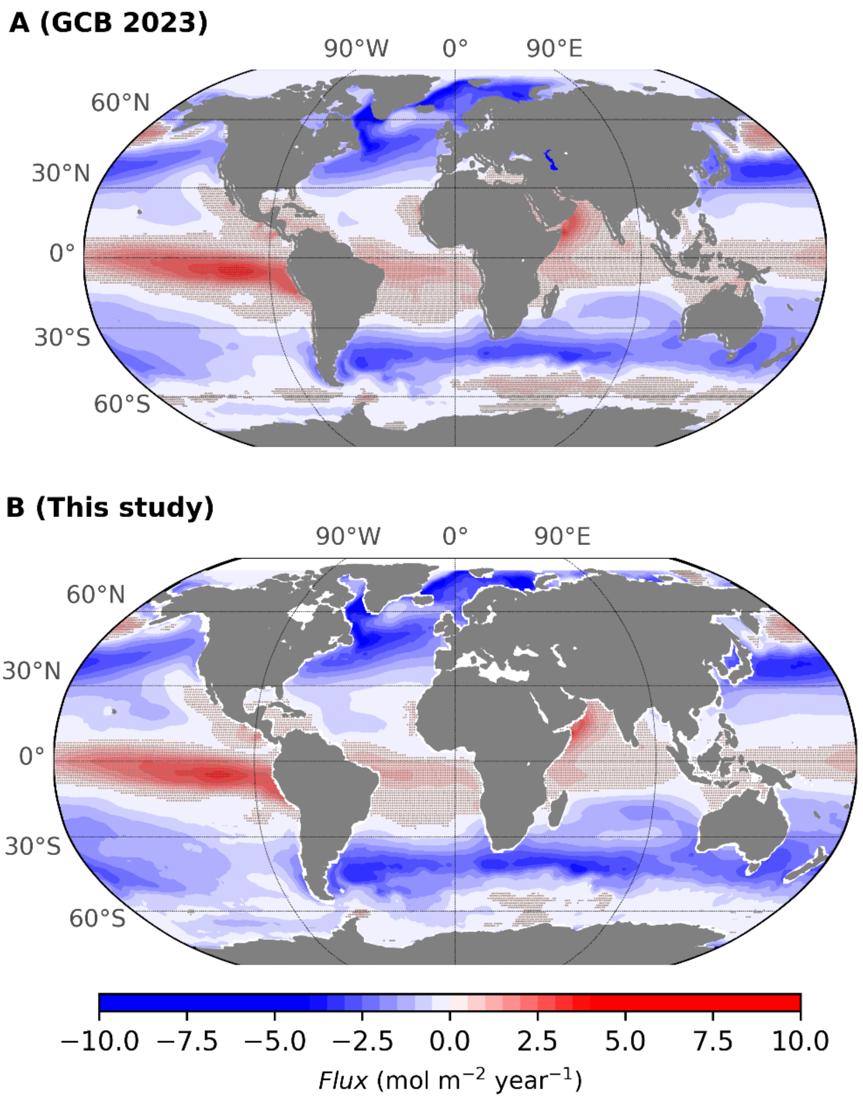

Fig. S10. Sea-air CO_2 flux in (A) the global carbon budget (GCB) 2023, and (B) this study. The flux in this study is based on the GCB 2023[9] with additional consideration of the asymmetric transfer and updated cool skin effect, as well as a warm bias effect (see Table 1 in the main text). Regions of CO_2 evasion into the atmosphere are hash-marked by grey dots.

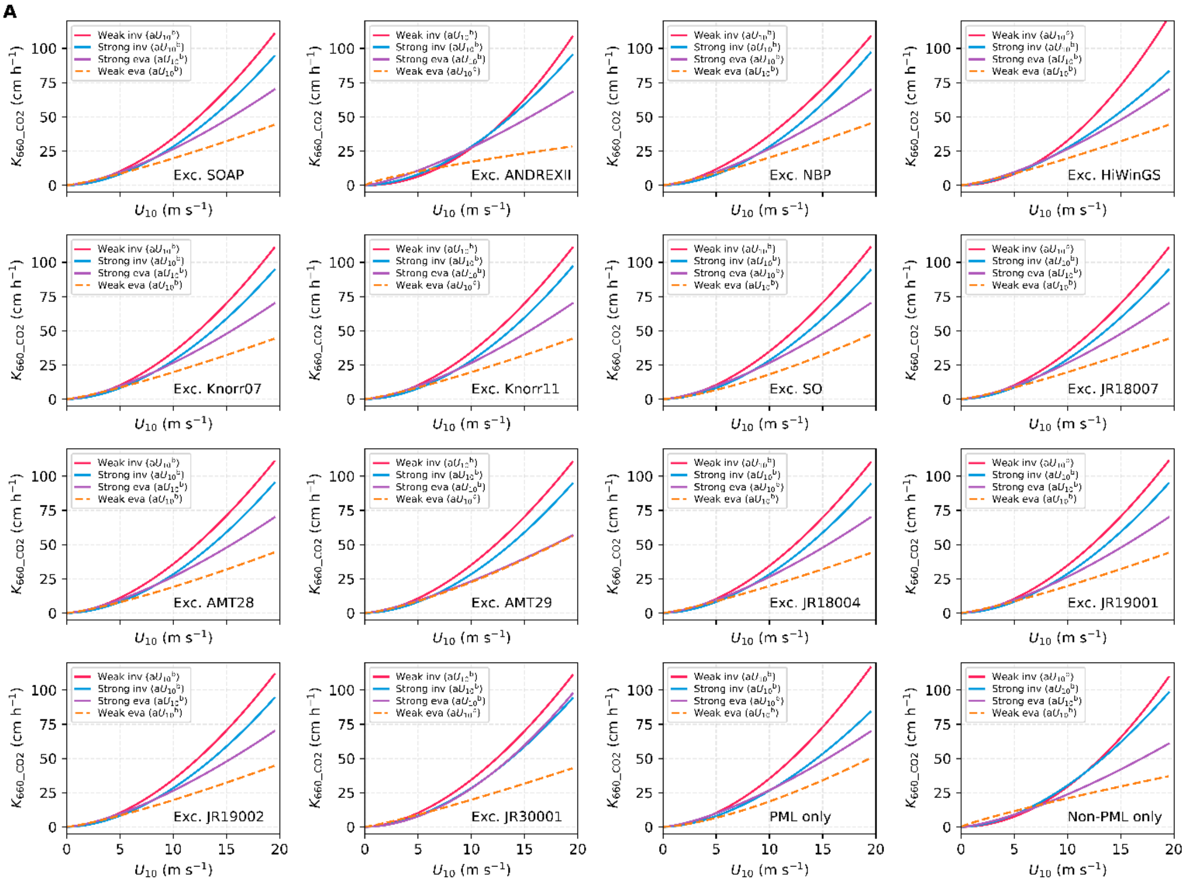

Fig. S11A. Parameterisations of gas transfer velocity as a function of U_{10}, excluding one or more cruises at a time, using the symmetric equation. The cruises ANDREXII, JR18007, AMT28, AMT29, JR18004, JR19001, JR19002, and JR30001 were all conducted by the Plymouth Marine Laboratory (PML) aboard two UK research vessels using consistent instrumentation and processing protocols[6].

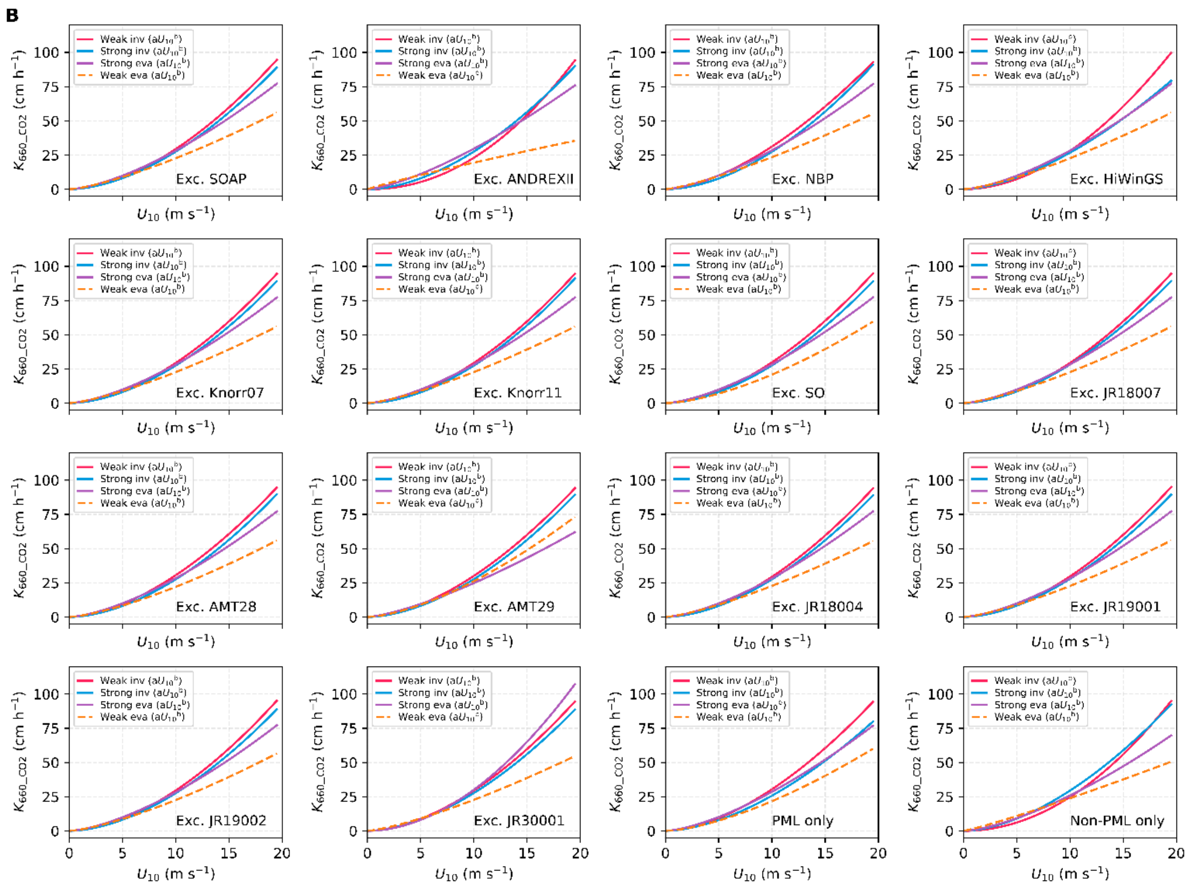

Fig. S11B. Parameterisations of gas transfer velocity as a function of U_{10}, excluding one or more cruises at a time, using the asymmetric equation.

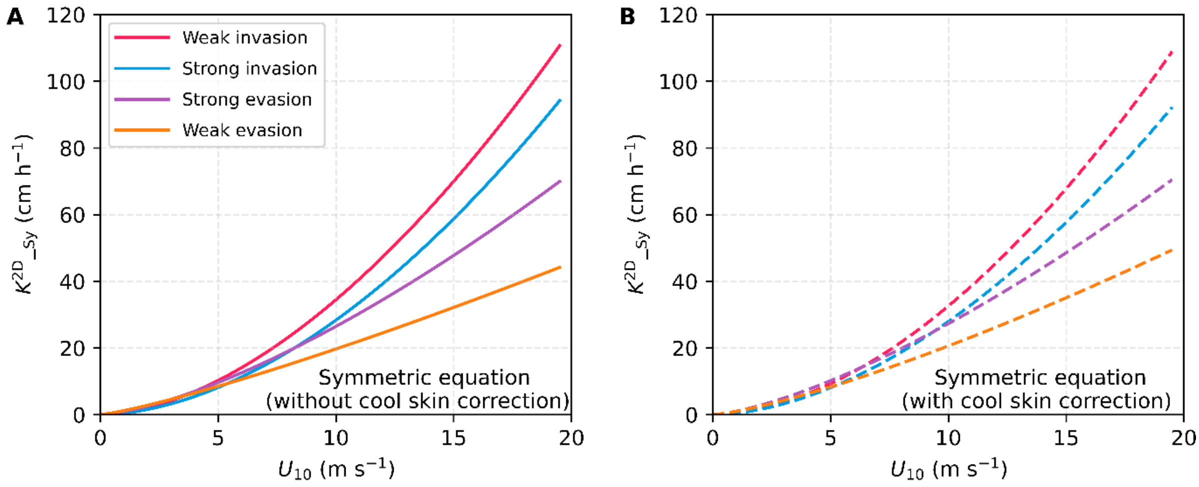

Fig. S12. Parameterisations of gas transfer velocity as a function of U_{10} with the 2D method. K^{2D} derived from the EC flux using the symmetric bulk equation without corrections of the cool skin effect (**A**) and with corrections of the cool skin effect (**B**).

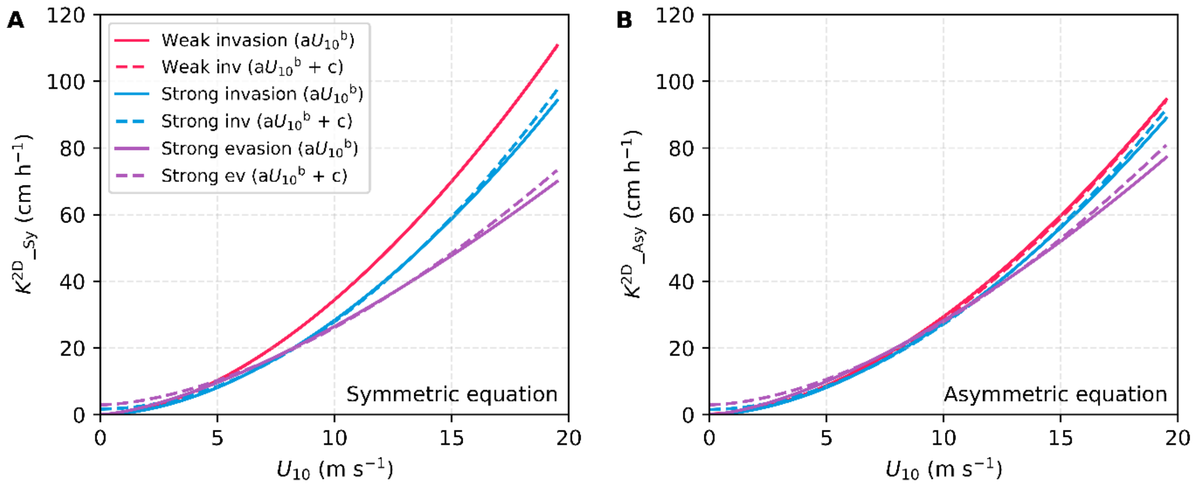

Fig. S13. Parameterisations of gas transfer velocity as a function of U_{10} with the 2D method. Solid lines: 2D fit formulation with zero intercept, i.e., $Flux = \Delta C_{660}(aU_{10}^{b})$; Dashed lines: 2D fit formulation with a non-zero intercept, i.e., $Flux = \Delta C_{660}(aU_{10}^{b} + c)$.

Table S1. Coefficients and R^2 of the 2D and 1D fits using the symmetric and asymmetric bulk equations with hourly data.

Fit type	Equation	Coefficient and R^2	Weak invasion $-20 < \Delta fCO_2 < 0$ µatm	Strong invasion $\Delta fCO_2 < -20$ µatm	Strong evasion $\Delta fCO_2 > 20$ µatm	Weak evasion $0 < \Delta fCO_2 < 20$ µatm	Strong signal $\lvert\Delta fCO_2\rvert > 20$ µatm	All data
2D fit	Symmetric bulk equation	*a*	0.62	0.45	0.93	1.22	0.45	0.45
		b	1.75	1.80	1.46	1.21	1.80	1.80
		R^2	0.26	0.63	0.41	0.014	0.69	0.70
	Asymmetric bulk equation	*a*	0.52	0.49	0.89	1.01	0.49	0.49
		b	1.75	1.75	1.50	1.35	1.75	1.75
		R^2	0.25	0.63	0.40	0.0091	0.69	0.71
1D fit	Symmetric bulk equation	*a*	-	0.44	0.70	-	0.44	-
		b	-	1.84	1.59	-	1.84	-
		R^2	-	0.59	0.43	-	0.59	-
	Asymmetric bulk equation	*a*	-	0.48	0.70	-	0.49	-
		b	-	1.78	1.63	-	1.78	-
		R^2	-	0.59	0.45	-	0.58	-

References

1. Blomquist, B. W. et al. Wind speed and sea state dependencies of air-sea gas transfer: Results from the High Wind Speed Gas Exchange Study (HiWinGS). *J. Geophys. Res. Oceans*, *122*(10), 8034–8062 (2017).
2. Fairall, C. W. et al. Air-sea trace gas fluxes: Direct and indirect measurements. *Front. Mar. Sci.*, *9*(7), 1–16 (2022).
3. Luhar, A. K., Woodhouse, M. T., & Galbally, I. E. A revised global ozone dry deposition estimate based on a new two-layer parameterisation for air-sea exchange and the multi-year MACC composition reanalysis. *Atmos. Chem. Phys.*, *18*(6), 4329–4348 (2018).
4. Leighton, T. G., Coles, D. G. H., Srokosz, M., White, P. R., & Woolf, D. K. Asymmetric transfer of CO_2 across a broken sea surface. *Sci. Rep.*, *8*(1), 1–9 (2018).
5. Yang, M. et al. Global synthesis of air-sea CO_2 transfer velocity estimates from ship-based eddy covariance measurements. *Front. Mar. Sci.*, *9*(6), 1–15 (2022).
6. Dong, Y., Yang, M., Bakker, D. C. E., Kitidis, V., & Bell, T. G. Uncertainties in eddy covariance air-sea CO_2 flux measurements and implications for gas transfer velocity parameterisations. *Atmos. Chem. Phys.*, *21*(10), 8089–8110 (2021).
7. Dong, Y. et al. Direct observational evidence of strong CO_2 uptake in the Southern Ocean. *Sci. Adv.*, *10*(30), eadn5781 (2024).
8. Wanninkhof, R. Relationship between wind speed and gas exchange over the ocean revisited. *Limnol. Oceanogr. Methods*, *12*(6), 351–362 (2014).
9. Friedlingstein, P. et al. Global Carbon Budget 2023*, Earth Syst. Sci. Data, 15*(12), 5301–5369 (2023).
